# Supplementary material for: REGALS: a general method to deconvolve X-ray scattering data from evolving mixtures
Source: IUCrJ. 2021 Feb 6;8(Pt 2):225–37. doi: 10.1107/S2052252521000555 (PMC7924237; doi:10.1107/S2052252521000555)
Supplement: Supplementary file 1 [file m-08-00225-sup1.pdf]

IUCrJ

Volume 8 (2021)

Supporting information for article:

REGALS: a general method to deconvolve X-ray scattering data from evolving mixtures

Steve P. Meisburger, Da Xu and Nozomi Ando

Supporting Information

REGALS: a general method to deconvolve X-ray scattering data from evolving mixtures

Meisburger *et al.*

Contents

Supplementary Tables **2**

Supplementary Tables

	**B1** (salt gradient)	**B2** (salt gradient)	**C1** (dimer)	**C2** (monomer)
**Concentration basis**				
Model	*smooth*	*smooth*	*smooth*	*smooth*
Range ($x_{\min}$–$x_{\max}$)[a]	1–1737	201–1737	730–1270	1150–1600
Control points	50	50	50	50
Zero boundary conditions	none	$x_{\min}$	$x_{\min}, x_{\max}$	$x_{\min}, x_{\max}$
Regularization parameter (λ')[b]	7.8×10^8	4.5×10^8	0	0
**SAXS basis**				
Model	*simple*	*simple*	*simple*	*simple*

[a] x = frame number
[b] Estimated using $n_g = 8$ (see Methods in the Main Text)

Table S1: REGALS model for *Bs*RNR AEX-SAXS dataset

	**C1** (resting tetramer)	**C2** (activated tetramer)	**C3** (unknown oligomer)
**Concentration basis**			
Model	*smooth*	*smooth*	*smooth*
Range ($x_{\min}$–$x_{\max}$)[a]	1–5	1–3.5	1–5
Control points	31	21	31
Zero boundary conditions	$x_{\min}$	$x_{\max}$	none
Regularization parameter (λ')	10	10	10
**SAXS basis**			
Model	*real-space*	*real-space*	*real-space*
$d_{\max}$ (Å)	130	130	300
Control points	101	101	101
Zero boundary conditions	$r = 0$, $d_{\max}$	$r = 0$, $d_{\max}$	$r = 0$, $d_{\max}$
Regularization parameter (λ)	1×10^{12}	1×10^{12}	1×10^{12}

[a] $x = \log_{10} c$ for $c > 0$ and $x = 1$ for $c = 0$, where c is the ligand concentration in μM

Table S2: REGALS model for PheH titration dataset

	C1 (dimer)	C2 (monomer)
**Concentration basis**		
Model	*smooth*	*smooth*
Range (x_{min}–x_{max})[a]	1.3–5.1	1.3–5.1
Control points	31	31
Zero boundary conditions	x_{max}	none
Regularization parameter (λ')	1×10^{-3}	1×10^{-3}
**SAXS basis**		
Model	*real-space*	*real-space*
d_{max} (Å)	70	62
Control points	101	101
Zero boundary conditions	$r = 0$, d_{max}	$r = 0$, d_{max}
Regularization parameter (λ)	1×10^{11}	1×10^{11}

[a] $x = \log_{10}(t)$ where t is the delay time in ms

Table S3: REGALS model for MsbA NDB time-resolved dataset

	C1 (transient)	C2 (intraparticle)	C3 (interparticle)
**Concentration basis**			
Model	*smooth*	*smooth*	*smooth*
Range (x_{min}–x_{max})[a]	2.75–6	2.75–6	2.75–6
Control points	31	31	31
Zero boundary conditions	x_{min}, x_{max}	none	none
Regularization parameter (λ')	10	10	10
**SAXS basis**			
Model	*real-space*	*real-space*	*real-space*
d_{max} (Å)	59	46	150
Control points	101	101	101
Zero boundary conditions	$r = 0$, d_{max}	$r = 0$, d_{max}	$r = 0$, d_{max}
Regularization parameter (λ)	1×10^{11}	1×10^{11}	1×10^{11}

[a] $x = \log_{10}(t)$ where t is the time delay in ns

Table S4: REGALS model for CypA T-jump dataset
